# 302. A Randomized Controlled Trial of Rapid Pathogen and Resistance Identification from Positive Blood Cultures

**DOI:** 10.1093/ofid/ofae631.092

**Published:** 2025-01-29

**Authors:** Shawn Vasoo, Christine B Teng, Pei Yun Hon, Regina SH Teng, Min Yi Yap, Sharon Syn Hui Wee, Jonathan Chia, Shehara M Mendis, Ezlyn Binti Izharuddin, Ray JH Lin, Po Ying Chia, Rees CS Sim, Charlene MZ Kang, Mark Chen, Angela Chow, Joanne Yoong, David Lye, Ritu Banerjee, Robin Patel, Paul Tambyah, Partha P De

**Affiliations:** National Centre for Infectious Diseases and Tan Tock Seng Hospital, Dept of Infectious Diseases, Singapore, Singapore; National University of Singapore; NCID, Singapore, Not Applicable, Singapore; NCID, Singapore, Not Applicable, Singapore; Tan Tock Seng Hospital, Singapore, Not Applicable, Singapore; Tan Tock Seng Hospital, Singapore, Not Applicable, Singapore; Tan Tock Seng Hospital, Singapore, Singapore, Singapore; Tan Tock Seng Hospital, Singapore, Not Applicable, Singapore; National Healthcare Group Polyclinics, Singapore, Not Applicable, Singapore; Tan Tock Seng Hospital, Singapore, Not Applicable, Singapore; National Centre for Infectious Diseases, Singapore, Not Applicable, Singapore; Tan Tock Seng Hospital, Singapore, Not Applicable, Singapore; National Centre for Infectious Diseases, Singapore, Not Applicable, Singapore; National Public Health & Epidemiology Unit (NPHEU), National Centre for Infectious Diseases, Singapore, Not Applicable, Singapore; Tan Tock Seng Hospital, Singapore, Not Applicable, Singapore; Research For Impact, Singapore; National University of Singapore, Singapore, Not Applicable, Singapore; National Centre for Infectious Diseases, Singapore, Not Applicable, Singapore; Vanderbilt University Medical Center, Nashville, TN; Mayo Clinic, Rochester, MN; National University Hospital, Singapore, Singapore, Not Applicable, Singapore; Tan Tock Seng Hospital, Singapore, Not Applicable, Singapore

## Abstract

**Background:**

Morbidity from bacteremia may be exacerbated by inadequate initial antibiotic therapy. This concern however may lead to hesitance to de-escalate while awaiting pathogen identification and susceptibility results. We performed a randomized controlled trial evaluating the impact of a strategy for rapid pathogen and resistance detection directly from positive blood culture bottles (BCBs) using a rapid multiplex PCR (rmPCR) complemented with a rapid phenotypic screen (RPS) for extended-spectrum beta-lactamases and carbapenemases in a setting with moderate to high levels of antimicrobial resistance where routine direct-from-BCB disk diffusion susceptibility testing was in place

**Methods:**

Patients with positive BCBs were randomized to 2 arms: 1) Standard BCB processing (including direct from BCB disk diffusion susceptibility testing - control, n=419) with antimicrobial stewardship (AS); or 2) rmPCR/RPS with AS (rmPCR/RPS, n=425). The primary outcome was time from positive blood culture result to prescription of an effective or optimal antibiotic regimen. An effective antibiotic regimen was defined as one active against the detected pathogen(s); an optimal antibiotic regimen was defined as an effective regimen which was also the most narrow spectrum and targeted one, as recommended by institutional guidelines. Mortality was analyzed as a secondary outcome.

**Results:**

True bacteremia was identified in 658 patients (n=329 in both arms); 597 were monomicrobial (405 with aerobic or facultative anaerobic Gram negatives rods [AGNR]) and 61 polymicrobial (41 harboring AGNR). Time from BCB Gram stain to prescription of an effective antibiotic was 9.9 versus 3.2 hours in the control and rmPCR/RPS groups, respectively (p=0.58) and time from Gram stain to prescription of an optimal antibiotic regimen was 41.6 versus 29.4 hours in the two groups, respectively (p < 0.001). 14-day mortality was 7.6% versus 4.3% (p = 0.069) and 30-day mortality was 11.2% versus 8.8% (p=0.30) in the two groups, respectively.

**Conclusion:**

Compared to control methods which included direct-from-BCB disk diffusion susceptibility testing, an intervention comprising rmPCR/RPS with AS applied to positive blood cultures improved time to optimal therapy but not time to effective therapy or mortality.

**Disclosures:**

**Shawn Vasoo, MBBS, MRCP, D(ABP), D(ABIM) (Inf Dis), FRCPath**, bioMerieux: In-kind, for this study|Rosco Diagnostica: In-kind, for this study **Robin Patel, MD**, a patent on Bordetella pertussis/parapertussis PCR issued, a patent on a device/method for sonication with royalties paid by Samsung to Mayo Clinic, a: See above|MicuRx Pharmaceuticals and BIOFIRE: Grant/Research Support|PhAST, Day Zero Diagnostics, Abbott Laboratories, Sysmex, DEEPULL DIAGNOSTICS, S.L., Netflix, Oxford Nanopore Technologies and CARB-X: Advisor/Consultant|Up-to-Date and the Infectious Diseases Board Review Course.: Honoraria **Paul Tambyah, MBBS (S'pore), Diplomate, American Board of Internal Medicine and Infectious Diseases**, Moderna: Grant/Research Support|Sanofi-Pasteur: Grant/Research Support

